# Emergency bedside cesarean delivery: lessons learned in teamwork and patient safety

**DOI:** 10.1186/1756-0500-5-412

**Published:** 2012-08-06

**Authors:** Michelle A O Kinney, Carl H Rose, Kyle D Traynor, Eric Deutsch, Hafsa U Memon, Staci Tanouye, Katherine W Arendt, James R Hebl

**Affiliations:** 1 Department of Anesthesiology, Mayo Clinic, Rochester, MN, USA; 2 Department of Obstetrics and Gynecology, Mayo Clinic, Rochester, MN, USA; 3 Mayo Clinic, 200 First Street SW, Rochester, MN, 55905, USA

**Keywords:** Perimortem cesarean section, Remifentanil, Pregnancy, Medication overdose

## Abstract

**Background:**

Maternal cardiovascular and pulmonary events during labor and delivery may result in adverse maternal and fetal outcome. Potential etiologies include primary cardiac events, pulmonary embolism, eclampsia, maternal hemorrhage, and adverse medication events. Remifentanil patient-controlled analgesia is an alternative when conventional neuraxial analgesia for labor is contraindicated. Although remifentanil is a commonly used analgesic, its use for labor analgesia is not clearly defined.

**Case presentation:**

We present an unexpected and unique case of remifentanil toxicity resulting in the need for an emergent bedside cesarean delivery. A 30-year-old G3P2 woman receiving subcutaneous heparin anticoagulation due to a recent deep vein thrombosis developed cardiopulmonary arrest during labor induction due to remifentanil toxicity.

**Conclusion:**

A rapid discussion among the attending obstetric, anesthesia, and nursing teams resulted in consensus to perform an emergent bedside cesarean delivery resulting in an excellent fetal outcome. During maternal cardiopulmonary arrest, a prompt decision to perform a bedside cesarean delivery is essential to avoid significant maternal and fetal morbidity. Under these conditions, rapid collaboration among obstetric, anesthesia, and nursing personnel, and an extensive multi-layered safety process are integral components to optimize maternal and fetal outcomes.

## Background

Emergent bedside cesarean delivery is an intrinsically unpredictable procedure performed for fetal indications following a catastrophic event or injury when maternal survival is uncertain. Under these conditions, a decisive and cohesive plan of action on the part of labor and delivery personnel, including anesthesia, obstetrics, nursing and neonatology, is linked to fetal and maternal prognosis [1]. We present a patient with a history of recent venous thromboembolism who experienced an unexpected cardiopulmonary arrest following implementation of an intravenous remifentanil patient-controlled analgesic (PCA) infusion for pain relief in labor and subsequently underwent an emergent bedside cesarean delivery.

## Case presentation

A 30-year-old G3P2 woman was admitted at 38^+6^ weeks of gestation for elective induction of labor due to concerns regarding intrapartum anticoagulation. The current pregnancy was complicated by an episode of idiopathic lower extremity deep venous thrombosis (DVT) at 10 weeks estimated gestation at which time an inferior vena caval (IVC) filter was placed. Following placement of the IVC filter, therapeutic anticoagulation was initiated with subcutaneous unfractionated heparin for the duration of the pregnancy. The remainder of her antecedent medical history was otherwise unremarkable.

Although the last injection of subcutaneous heparin was administered 12 h before hospital arrival, the patient’s activated partial thromboplastin time (aPTT) was significantly elevated at 70 s (normal range: 21-33 s) at the time of admission. The patient was subsequently counseled that neuraxial analgesia was contraindicated in the setting of an elevated aPTT due to an increased risk of neuraxial bleeding. The decision was made to ripen the cervix with misoprostol and administer intermittent intravenous (i.v.) fentanyl as needed for labor analgesia. The fetal heart rate pattern remained reassuring throughout this period of time. A repeat aPTT obtained 6 h later remained elevated at 80 s. After discussing potential options with the patient, it was decided to proceed with i.v. remifentanil patient-controlled analgesia (PCA) based on our institutional protocol (basal infusion: 0.03-0.05 μg/kg/min; bolus dose 0.2-0.8 μg/kg every 5 min). A basal infusion rate of 0.04 μg/kg/min (2 μg/min) with a bolus dose of 0.8 μg/kg (40 μg) every 5 min was ordered based on her ideal body weight of 50 kg. The medication was prepared by the hospital pharmacy and placed in a barcode syringe for administration in a Hospira LifeCare® Infusion System which was connected to an 18-gauge cannula sited in the woman’s left hand.

The remifentanil PCA was initiated after appropriate medication review by anesthesia and nursing personnel, confirming the drug, concentration, dose, and route of administration and initiation of pulse oximetry. The mother was supine, with left uterine displacement. Within seconds of administering the first PCA dose, the patient stated that she could not open her eyes and rapidly became rigid and apneic. Her oxygen saturation (SpO2) decreased to approximately 80%, her pulse declined from 97 to 57 beats/min, and her blood pressure fell from 126/74 to 92/57 mmHg. Anesthesia personnel immediately performed a jaw-thrust maneuver to open the patient’s airway and initiated bag-mask ventilation with 100% oxygen at 15 L/min using a flow inflating resuscitation bag. Simultaneous to these events, the hospital code team was summoned by nursing personnel. The remifentanil infusion was immediately disconnected and an i.v. bolus of normal saline 500 mL administered. Two-handed bag-mask ventilation was difficult due to maternal rigidity, but the patient’s SpO2 improved to >90% within three minutes. Despite appropriate resuscitative efforts and maintenance of left uterine displacement, fetal bradycardia occurred. Subsequently, the maternal pulse (radial and femoral) was undetectable and fetal cardiac activity was simultaneously lost. Immediately before initiating cardiopulmonary resuscitation (CPR), the maternal pulse returned, precluding the need for maternal chest compressions. A fetal scalp electrode did not detect the fetal heart rate. Faculty members from the Departments of Anesthesiology and Obstetrics rapidly conferred and decided to proceed with an emergent bedside cesarean delivery given the ongoing difficulty with two-handed bag-mask ventilation, non-reassuring fetal status, and concern for further delay if the patient was transported to an operating room. The differential diagnosis included massive pulmonary embolus, amniotic fluid embolus, intracranial hemorrhage, a primary cardiac event, or adverse medication reaction.

An emergent bedside classical cesarean delivery was performed through a low midline vertical incision 4 min after the loss of fetal cardiac activity and 5 min after loss of maternal cardiac output. Delivery of a term female infant with Apgar scores of 1 and 8 at 1 and 5 min, respectively, followed. Umbilical cord arterial gas analysis showed a respiratory acidosis with pH 7.07, CO_2_ 89 mmHg, O_2_ 13 mmHg, HCO_3_ 25 mmol/L, and base deficit of -4 mmol/L. Intravenous etomidate 20 mg and succinylcholine 120 mg were administered at the time of surgical incision to facilitate maternal tracheal intubation. There was no patient movement with surgical incision. Following delivery, the patient’s blood pressure was 136/74 mmHg, her pulse was 135 beats/min, and her SpO_2_ was 98%. Initial maternal arterial blood gas results obtained 6 min after delivery of the infant were: pH 7.17, CO_2_ 57 mmHg, O_2_ 192 mmHg, HCO_3_ 21 mmol/L, and base deficit -7 mmol/L. Additional i.v. medications included midazolam 4 mg, ketamine 20 mg, fentanyl 150 μg, and propofol 40 mg. Maternal pulse decreased to 71 beats/min and her SpO_2_ remained >90% for the remainder of her operation.

The hysterotomy and abdominal wall were temporarily closed in the labor room and the patient transferred to the operating room where the abdominal cavity was inspected. Sevoflurane and oxygen were administered, as were intravenous oxymorphone 1.4 mg, meropenem 500 mg, and ondansetron 4 mg. After extensive irrigation, the hysterotomy and abdominal incision were closed using a standard technique. Following closure of the skin incision, the patient was considered sufficiently stable for extubation and was taken to the postoperative recovery room for extended monitoring. The infant was transferred to the intermediate care nursery for observation. The patient’s postpartum recovery was complicated by wound separation with healing by secondary intention. The patient had no explicit recall of the intraoperative surgical events and she had complete neurologic recovery with no deficit.

Pharmacy personnel immediately began investigating the possibility of an adverse medication event. The remifentanil infusion bag and PCA apparatus were collected and secured as per protocol. Within 3 h of the event, a medication error was confirmed; the compounded remifentanil solution had a concentration ten-fold higher than was labeled on the medication syringe. As a result, the patient had received an i.v. bolus dose of remifentanil 400 μg rather than the 40 μg bolus dose ordered. Following an extensive root cause analysis and patient event review, Pharmacy Services instituted several safeguards and modifications to its compounding protocols to significantly reduce the risk of future compounding errors. Specifically, only one concentration of remifentanil is now prepared, and the instructions for preparation are available on the pharmacy computer without any mathematical computation required.

## Conclusion

During the period 1985-2004, traumatic and cardiac events were the most common causes of maternal cardiac arrest and associated perimortem cesarean delivery [2]. Additional causes included embolism, magnesium overdose, sepsis, anesthetic events, eclampsia, spontaneous uterine rupture, and intracranial hemorrhage. Fetal, and possibly maternal, outcomes appear optimized by timely delivery. Cardiopulmonary resuscitation is generally less effective in the third trimester because of aortocaval compression. Emptying the gravid uterus may ensure better survival of the infant and also allow for more successful maternal resuscitation; particularly if the reason for the cardiac arrest is reversible. Published reports support the long-standing recommendation to initiate a perimortem cesarean delivery within four minutes of maternal cardiopulmonary arrest; with delivery of the infant within five minutes. This potentially allows for maternal cerebral oxygenation to be reestablished to prevent or reduce maternal neurologic damage [2].

The effect of perimortem cesarean section on maternal circulation in 20 reported cases from 1985-2004 demonstrated the return of spontaneous circulation or improvement in hemodynamic status in 12 (60%) mothers. There was no change in the hemodynamic status in eight (40%) mothers where the cause of arrest was lethal [2]. In our patient, cesarean delivery was started within four minutes of the loss of the fetal heart trace, and less than one minute elapsed before delivery. Similar to other reports, maternal hemodynamics in our case dramatically improved immediately following delivery.

Although our patient was *not* undergoing chest compressions at the time of delivery, maternal respiratory arrest, difficult bag-mask ventilation with associated hypoxemia, profound maternal bradycardia, and loss of fetal cardiac activity precluded transport to an operating room. Our management strategy was guided, in part, by recently published data from the Anesthesia Patient Safety Foundation (APSF) on maternal cardiac and respiratory arrests [3]. Upon reviewing anesthesia malpractice claims associated with maternal cardiopulmonary arrests, only 1 (5%) of 22 patients experienced survival without sequelae or permanent neurologic injury. In that case, two unique features existed, (1) an anesthesiologist was present to provide immediate airway management; and (2) the cesarean delivery was performed in the labor room within minutes of maternal arrest. Importantly, transfer of an unstable patient to an operating room for better intubating or surgical conditions was not performed, and is currently not recommended [4].

The etiology of our patient’s cardiopulmonary arrest was acute remifentanil toxicity. Remifentanil is an ultrashort-acting synthetic opioid compound with an ester link that confers susceptibility to rapid hydrolysis by nonspecific tissue and blood esterases, and has a very short context-sensitive half-life of three minutes [5]. Remifentanil is highly lipid soluble and rapidly crosses the placenta. The mean remifentanil umbilical vein: maternal artery concentration ratio of 0.88 suggests significant placental transfer of remifentanil. Remifentanil undergoes rapid redistribution and/or metabolism within the fetus, with an umbilical arterial to umbilical venous concentration ratio of 0.29 [6]. In this case, the infant’s 1- and 5- minute Apgar scores improved dramatically from 1 to 8 exclusively with positive pressure ventilation, without the need to administer naloxone or other medications.

During our patient’s event, consideration was given to an empiric administration of naloxone. However, because the primary etiology of the maternal arrest was unknown at the time, the potential benefit to the mother and baby was uncertain. The complete loss of fetal cardiac activity suggested that a further delay in delivery may have resulted in a severely depressed neonate and a difficult resuscitation. Furthermore, the history of maternal venous thromboembolism increased the likelihood of a life-threatening embolic etiology. An i.v. dose of naloxone 0.4 mg would have been unlikely to result in significant improvement in either maternal or fetal condition given the estimated dose of remifentanil administered [7]. Naloxone administration in these circumstances may have only complicated perioperative maternal management and could have harmed the fetus by delaying delivery and resuscitation. Our patient’s cardiopulmonary status rapidly improved following delivery, suggesting a reversible, transient etiology rather than an embolic event. In retrospect, our patient likely had extreme bradycardia with respiratory arrest and rigidity of her chest wall due to remifentanil toxicity. Complete cardiac arrest was therefore unlikely.

Rapid teamwork and a multidisciplinary consensus regarding clinical management are essential components for optimal maternal and fetal outcome during adverse maternal events. Routine practice drills and the creation of dedicated perimortem cesarean equipment packs have been recommended by some authors [8]. In our case, an emergency perinatal paging system was activated by our unit secretary to immediately summon obstetricians, labor nurses and the obstetric anesthesia and pediatric teams. Our perinatal group paging system was designed by Dr. Paula Craigo with the intention of decreasing the time spent paging multiple care providers, thereby expediting patient care for an emergent cesarean delivery [9]. Any member of the perinatal team can call our hospital operator and request perinatal group pager activation. In this case, ten team members assembled emergently: an obstetrician, two obstetrical resident physicians, one anesthesiologist, one nurse anesthetist, one anesthesiology resident physician, two obstetrical nurses, one pediatric nurse, and one pediatric physician resident. One obstetric nurse readied the surgical equipment, another obstetric nurse stayed with the patient’s husband in a waiting room and provided emotional support, the obstetricians performed the surgery, the anesthesia providers managed the patient’s airway, and induced and maintained the anesthetic, while the pediatrics team readied their equipment in the infant resuscitation room and resuscitated the baby. In addition, a hospital-wide emergency cardiorespiratory arrest group pager system was also activated by the unit secretary, with response from six additional team members: a senior anesthesia resident who supplied additional medications, an intensive care nurse who placed an additional intravenous line, a respiratory therapist who placed the arterial line, and three pharmacists who charted vital signs and the medications that were administered, and who also participated in the immediate investigation regarding the cause of the incident. The teamwork was organized, with individuals performing their usual role. The anesthesiologist and obstetrician each led their respective teams, and also communicated and worked together, calmly sharing decisions such as when to transfer the patient to the operating suite.

Surgical instruments were obtained by an obstetric nurse from the operating suite approximately 30 meters away from the patient’s room. Equipment and medications to facilitate endotracheal intubation were similarly obtained by the anesthesiologist from the anesthesia workroom 30 meters away. Basic airway management supplies were located in each labor room in a plastic box in a cupboard. These supplies were utilized, including in-wall oxygen access and tubing, a flow-inflating ventilation bag and mask, and suction equipment.

Remifentanil dosing regimens vary, with patient controlled boluses ranging from 0.2-1 μg/kg, and infusions of 0.025-0.1 μg/kg/min [10]. Standardized medication utilization processes for i.v. remifentanil PCA administration at our institution include electronic ordering protocols, concentration and dispensing preparation, and integration of ‘smart’ infusion pumps with barcode technology and drug library software. In addition, institutional safety protocols require (1) nursing and anesthesia team members to be present upon initiation of a remifentanil PCA; and (2) oxygen saturation to be continuously monitored by pulse oximetry. All of the aforementioned safety features were in place at the time of our patient’s care and likely contributed to the immediate recognition and successful management of compromised maternal status. Finally, our institution subsequently adopted a policy of conducting twice daily “Safety Rounds” to familiarize staff with hospitalized patients and individual care responsibilities.

The relative importance of each of these safety practices remains unknown; however, their integration into practice should facilitate immediate maternal resuscitation in conjunction with rapid teamwork and a multidisciplinary approach to patient care, all of which are essential components to overcome adverse maternal and fetal events.

## Consent

Written informed consent was obtained from the patient for publication of this case report. A copy of the written consent is available for review by the Editor-in-Chief of this journal.

## Abbreviations

PCA: Patient-controlled analgesia; DVT: Deep venous thrombosis; IVC: Inferior vena cava; aPTT: Activated partial thromboplastin time. i.v., intravenous; CPR: Cardiopulmonary resuscitation; APSF: Anesthesia Patient Safety Foundation.

## Competing interests

The authors declare that they have no competing interests. For all the authors, the only source of funding for this manuscript was Mayo Clinic.

## Authors’ contributions

MAOK conceived the case report, participated in the care of the patient and the sentinel event committee, and drafted and revised the manuscript. CHR participated in the care of the patient, and drafted and revised the manuscript. KDT participated in the care of the patient and contributed to manuscript preparation. ED participated in the care of the patient, and drafted and revised the manuscript. HUM participated in the care of the patient and contributed to manuscript preparation. ST participated in the care of the patient and contributed to manuscript preparation. KWA participated in root cause analysis, and drafted and revised the manuscript. JRH participated in the care of the patient, participated in the sentinel event committee and root cause analysis, and revised the manuscript. All authors read and approved the final manuscript.
